# Breaking It Down: The Ubiquitin Proteasome System in Neuronal Morphogenesis

**DOI:** 10.1155/2013/196848

**Published:** 2013-02-14

**Authors:** Andrew M. Hamilton, Karen Zito

**Affiliations:** Center for Neuroscience, University of California Davis, 1544 Newton Court, Davis, CA 95618, USA

## Abstract

The ubiquitin-proteasome system (UPS) is most widely known for its role in intracellular protein degradation; however, in the decades since its discovery, ubiquitination has been associated with the regulation of a wide variety of cellular processes. The addition of ubiquitin tags, either as single moieties or as polyubiquitin chains, has been shown not only to mediate degradation by the proteasome and the lysosome, but also to modulate protein function, localization, and endocytosis. The UPS plays a particularly important role in neurons, where local synthesis and degradation work to balance synaptic protein levels at synapses distant from the cell body. In recent years, the UPS has come under increasing scrutiny in neurons, as elements of the UPS have been found to regulate such diverse neuronal functions as synaptic strength, homeostatic plasticity, axon guidance, and neurite outgrowth. Here we focus on recent advances detailing the roles of the UPS in regulating the morphogenesis of axons, dendrites, and dendritic spines, with an emphasis on E3 ubiquitin ligases and their identified regulatory targets.

## 1. Introduction

Ever since the ubiquitin proteasome system (UPS) was first characterized in the mid-20th century as the primary mediator of regulated protein degradation, its role in neurons has come under ever increasing scrutiny. Due to the large distances separating many synapses from the soma, local protein synthesis and degradation are particularly important to neuronal development and function. The diverse neuronal processes subject to regulation by the UPS range from long-term potentiation and homeostatic plasticity to acute regulation of neurotransmitter release. Several comprehensive reviews have been published on the importance of the UPS in synaptic plasticity [1, 2], intracellular trafficking [3, 4], and disease states [5, 6]; this paper will focus on the UPS-dependent regulation of neuronal morphogenesis.

## 2. The Ubiquitin Proteasome System

**Figure 1:**
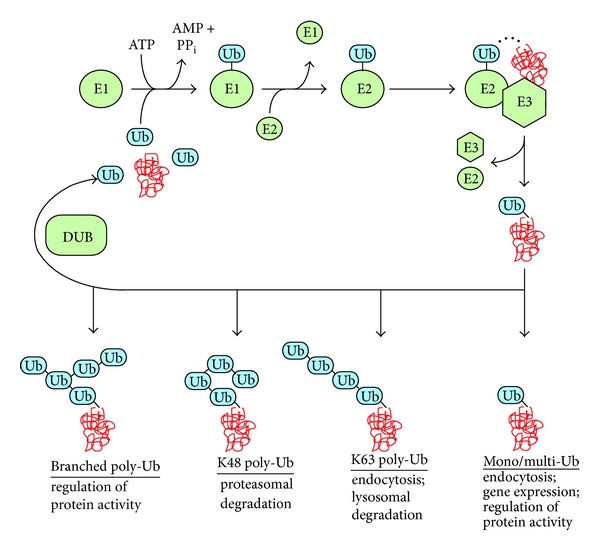
Ubiquitination and ubiquitin-mediated trafficking. Ubiquitin (Ub) is activated in an ATP-dependent manner by an E1, passed to an E2 ubiquitin conjugase, and finally transferred to a target protein by an E2/E3 ubiquitin ligase complex. Following monoubiquitination, the addition of further ubiquitin moieties occurs at specific lysine residues and results in one of a variety of polyubiquitin chains, each possessing a unique set of known consequences for protein regulation and trafficking. The ubiquitination state of a protein is regulated both via the addition of ubiquitin and also via the removal of single moieties or chains by deubiquitinases (DUBs).

Ubiquitin, aptly named for its intracellular omnipresence, is a small 76 residue protein which may be tagged onto target proteins as single moieties or polyubiquitin chains (Figure 1). Ubiquitination most famously serves to regulate protein degradation via the action of the ubiquitin proteasome system. In addition, ubiquitination has been shown to regulate a diverse array of cellular processes, including endocytosis, DNA repair, cell division, and protein trafficking [7, 8]. Ubiquitin is initially charged in an ATP-dependent manner by an E1 activating enzyme and then transferred to an E2 ubiquitin conjugating enzyme. The Ub-E2 interacts with an E3 ubiquitin ligase, and this Ub-E2-E3 complex attaches the activated ubiquitin to a specific target through the carboxy-terminal glycine of ubiquitin. Additional ubiquitin ligands may then be bound to the previously attached ubiquitin moieties through one of 7 internal lysine residues on the ubiquitin itself.

 Multiple rounds of ubiquitination may result in a polyubiquitin chain, whose functional consequence depends on its three-dimensional structure, as conferred by the internal lysines used to link the chain together [8]. While any of the 7 ubiquitin lysines (K6, K11, K27, K29, K33, K48, or K63) may, in theory, be used to create a polyubiquitin chain, the results of K-48 and K-63 chains have been the best characterized [7, 9]. K-48 polyubiquitination directs proteins to the 26S proteasome, a massive proteolytic complex, where proteins are broken down into small oligopeptides and recycled. K-63 polyubiquitination, on the other hand, directs the endocytosis and lysosomal degradation of membrane proteins. Other forms of mono- or polyubiquitination have been shown to regulate protein processing, activity, or localization, rather than destruction [3, 8].

 While all cells make extensive use of the UPS, neurons have developed the remarkable ability to rapidly regulate the proteasome in response to changes in synaptic activity. Not only is the proteasome necessary for activity-dependent regulation of key synaptic proteins such as scaffolding proteins and neurotransmitter receptors [10–13], direct pharmacological stimulation or inhibition of neural activity alters proteasomal localization [14–16] and activity level [15, 17] in a matter of minutes. Furthermore, activity-dependent changes in proteasomal degradation occur in what appears to be a highly specific manner [10], suggesting precise regulatory mechanisms for targeting of individual synaptic proteins by the UPS. The intricacy of UPS regulation in neurons has engendered intense interest in how ubiquitination and protein degradation contribute to neuronal development and function. This paper focuses on the role of the UPS in neuronal morphogenesis, particularly in the development of axons, dendrites, and dendritic spines.

## 3. Regulation of Axonal Growth and Guidance by the UPS

One of the vital steps in the establishment of neural circuits, the growth and guidance of axons, has been shown to be regulated by the UPS in a number of model organisms. The best characterized targets of ubiquitination and proteasomal degradation in axons comprise proteins involved in regulating the axonal cytoskeleton, affecting both microtubules and actin filaments. These target proteins range from transcriptional factors to small GTPases, highlighting the wide reach of the UPS in neuronal cell biology.

### 3.1. Axon Growth

The UPS was first linked to axonal growth in the early 2000s, when mutants of the PHR family of E3 ubiquitin ligases were identified in screens for neuronal morphology defects in both *D. melanogaster* and *C. elegans*. The *highwire* mutant in *Drosophila* was isolated as a result of altered morphology of the neuromuscular junction (NMJ), displaying increased numbers of presynaptic boutons and greater bouton size and axonal branching [18]. Shortly afterwards, it was conclusively demonstrated that Highwire, an E3 ubiquitin ligase, regulates synaptic function and axon morphology through the ubiquitin proteasome system, as mutation of the deubiquitinase *fat facets* represses the *highwire* mutant phenotype [19]. Concurrently, mutations in RPM-1, Highwire's homolog in *C. elegans*, were shown to result in disorganized synapse morphology and axon overshoot and inappropriate pathfinding during development [20, 21]. This regulation of axonal morphogenesis occurs presynaptically, as RPM-1::GFP localizes to presynaptic terminals [21], and rescue of the *rpm-1* mutant phenotype occurs only with reintroduction of RPM-1 to the presynaptic neuron [20]. These findings, the first of many, proved that the role of the UPS in regulating axon outgrowth is highly conserved throughout the animal kingdom.

 A big surprise came several years later, when the anaphase promoting complex (APC), a classical component of the cell cycle [22], was discovered to be both highly expressed in postmitotic neurons and localized to synapses [23–25]. This massive E3 complex, composed of at least 12 subunits, requires binding by Cdh1 or Cdc20 to function, allowing APC activity to be controlled in distinct subcellular compartments by either of these activator proteins [26]. The APC, specifically in conjunction with Cdh1, regulates axon length [25, 27, 28]. Either RNAi knockdown of Cdh1 or inhibition of overall APC function causes an increase in axon length, both in cultured neurons [25, 28, 29] and *in vivo* [25]. The necessity of Cdh1-APC in axon growth is nuclear in nature, as rescue of Cdh1 knockdown is prevented by tagging the reintroduced Cdh1 with a nuclear export signal [29], while nuclear export and subsequent degradation of Cdh1 result in increased axon growth [30]. 

Regulation of axon growth by Cdh1-APC within the nucleus depends on the degradation of at least 2 separate transcription factors: Id2 [28] and SnoN [27, 29]. Id2 is ubiquitinated and degraded in an APC-dependent manner during developmental periods of intense axogenesis, while Id2 overexpression increases axon length [28]. The mode of Id2 action seems to be the repression of bHLH transcription factors, such as E47, which themselves suppress axon growth [28]. On the other hand, SnoN enhances the transcription of actin binding protein Ccd1, a positive regulator of axon growth through the JNK signaling pathway [27]. Knockdown of Ccd1 represses axon growth and completely blocks the axon stimulatory effect of SnoN overexpression. Furthermore, degradation of SnoN may require the transcriptional factors Smad2/3, as Smad2/3 interact directly with SnoN, while Smad2 RNAi increases axon length in an SnoN-dependent manner [31]. Thus, the APC acts as a brake on axon growth indirectly, by regulating the stability of transcription factors that either work to enhance or derepress axon growth.

 Another E3 that plays a role in both dendritic and axonal morphogenesis is Nedd4. Nedd4 is particularly intriguing due to the observation that Nedd4 is released from intramolecular autoinhibition by binding to calcium [32]. As intracellular calcium influx is a hallmark of robust synaptic activity, it may be that Nedd4-1 is activated during synaptic plasticity. During *Xenopus* embryonic development, direct inhibition of proteasomal degradation through ubiquitin-K48R overexpression or knockdown of Nedd4-1 results in decreased axon branching in the tectum [33]. The same group found that Nedd4-1 knockdown increases the levels of the lipid phosphatase PTEN in growth cones and that loss of PTEN rescues the effects of Nedd4-1 knockdown. The potential for Nedd4 regulation of PTEN is supported by the finding that Nedd4-1 interacts with and ubiquitinates PTEN [34]. Finally, Nedd4-1 RNAi decreases neurite outgrowth and branching in young dissociated cultures in a PTEN-dependent manner [35], suggesting that Nedd4-1-dependent regulation of the PI3 K signaling pathways downstream of PTEN is necessary for normal axon outgrowth. In addition to PTEN, Nedd4-1 regulates the trafficking of a variety of membrane proteins, including voltage-gated calcium channels [36], the FGF receptor tyrosine kinase [37], and the AMPA class of glutamate receptors [13, 38]. Also of note, a nonneural paralog of Nedd4-1 (Nedd4L) has been shown to target the transcription factors Smad2/3 in heterologous cell lines [39], similar to the APC [31]. This finding, however, has not yet been recapitulated in neural tissue. Based on its wide-reaching influence and sensitivity to calcium influx, Nedd4 has the potential to emerge as a key regulator of activity-dependent neuronal morphogenesis.

While the PHR proteins, the APC, and Nedd4-1 are the most well-studied UPS-dependent regulators of axon growth, the E3 ligases Smurf1, KLHL20, and Rnf6 also have identified roles. Notably, they also act through regulation of the actin cytoskeleton. Smurf1 has been shown to regulate axon outgrowth through RhoA, a small GTPase associated with the contraction of filamentous actin networks. Smurf1 directly targets RhoA for ubiquitination and proteasome-dependent degradation [40], which increases filopodial outgrowth in heterologous cell culture [40] and enhances axonal outgrowth and differentiation in cultured neurons [41]. In addition, Smurf1 itself is degraded by the proteasome and ubiquitinated by nuclear Cdh1-APC [42]. This process does not appear to be a part of the Cdh1-APC pathways involving the SnoN and Id2 transcription factors, as Smurf1 knockdown only partially abrogates the axonogenic effects of SnoN or Id2 overexpression [27–29, 42]. Reduced axon length induced by Smurf1 knockdown is rescued by overexpression of Smurf1 constructs tagged with either nuclear localization or exclusion sequences, suggesting that Smurf1 could act both within the nucleus and the cytoplasm [42]. In a similar manner to Smurf1, Rnf6 and KLHL20 indirectly target the actin cytoskeleton. KLHL20, a SKP-Cullin-F-Box (SCF) E3 complex, was found to ubiquitinate the RhoA activator RhoGEF [43]. KLHL20 knockdown decreases axon length and increases the incidence of growth cone collapse, while both effects are blocked by knocking down RhoGEF expression [43]. Rnf6, in a slightly less direct manner, ubiquitinates and enhances the degradation of LIM kinase 1 [44]. Overexpressing LIMK1 and knocking down Rnf6 both increase axon length in cultured neurons, while overexpression of Rnf6 decreases LIMK1 content at growth cones [44]. Thus, Rnf6 may act as a brake on growth cone motility by downregulating LIMK1, which has been shown to increase actin dynamics by phosphorylating ADF/cofilin [45].

### 3.2. Axon Guidance

While members in the PHR family of E3 ligases can regulate axon growth, their primary role appears to be the regulation of axon guidance. PHR members include PAM (human) and Phr1 (vertebrates), Highwire (*D. melanogaster*), and RPM-1 (*C. elegans*). The effects of manipulating PHR family members across species are remarkably similar, suggesting a conserved role in regulating axonal morphogenesis during neural development.

*rpm-1* mutant worms were first identified for their axonal defects, including defective presynaptic formation [21], axon guidance [20], and axon overgrowth [20]. The known functions of RPM-1 are split between direct regulation of the cytoskeleton and control of membrane protein trafficking. First, RPM-1 was found to interact genetically and biochemically with Rab activator GLO-4 and kinase DLK-1, both of which are necessary for the axon overextension phenotype [46]. The RPM-1 phenotype was rescued by *dlk-1* mutants, while DLK-1 itself was shown to be ubiquitinated and degraded by RPM-1 [46, 47]. It was demonstrated, however, that DLK-1 and GLO-4 do not interact genetically [46], indicating that RPM-1 takes part in the regulation of both actin dynamics, through GLO-4, and microtubules, through DLK-1. Second, the axon overgrowth and misguidance phenotype in *rpm-1* mutants can be suppressed by mutations in UNC-5 (Netrin receptor) or SAX-3 (Nogo receptor), while localization of both GFP-tagged UNC-5 and SAX-3 receptors is altered in *rpm-1* mutants [48]. Thus, RPM-1 may influence axon guidance by regulating the trafficking of Netrin and Nogo receptors. Finally, RPM-1 function has been shown to depend on interaction with several accessory proteins. RPM-1 binds to both the WD40 repeat protein RAE-1 [49] and the ubiquitin E2 variant UEV-3 [50]. RAE-1 interacts genetically with other RPM-1 binding proteins such as GLO-4 and results in a similar axon overgrowth phenotype to RPM-1 [49], while loss of UEV-3 function represses the RPM-1 phenotype, possibly through its interactions with elements of the MAPK signaling cascade such as DLK-1 and PMK-3 [50]. The transcription factor CEBP-1 and a number of MAP kinases and MAPK interacting proteins have also been shown as necessary for the RPM-1 phenotype [51], as well as the F-box protein FSN-1 [52]. In total, these results indicate that RPM-1 plays a role in a variety of pathways regulating axon guidance, including the trafficking of membrane proteins, transcription, and regulation of the actin and microtubule-based cytoskeleton.

 Following its discovery as a mutation that resulted in altered morphology of the Drosophila neuromuscular junction [18, 19], it has been demonstrated that *highwire* mutants also display aberrant axon guidance in the Drosophila olfactory system [53], indicating the importance of HIW in both the central and peripheral nervous systems. As with RPM-1, HIW function also depends on its association with Rae1, as the two proteins interact biochemically and genetically, and display similar mutant phenotypes [54]. Rae1 also appears to protect Highwire from degradation by autophagy, as Rae1 mutation decreases HIW protein levels, while this reduction can be partially blocked through mutation of essential lysosomal proteins [54]. The *hiw* synaptic overgrowth and axon guidance phenotypes both depend on the action of the Drosophila homolog of DLK-1, Wallenda, as its mutation suppresses the *hiw* phenotype [53, 55]. Mutation of a vital component of the HIW E3 ligase complex (*DFsn*) results in elevated levels of Wallenda and phenocopies the *hiw* mutant, suggesting that ubiquitination of Wallenda by Highwire may control elements of axon morphogenesis [56].

 Similarly to invertebrates, loss of Phr1 function in mice and zebrafish results in axon pathfinding defects, including incorrect mapping of retinal axons to the thalamus [57, 58], tectum [59], and superior colliculus [60], as well as gross anatomical abnormalities in the major axon tracts of the hippocampus, thalamus, and corpus callosum [57]. Furthermore, axons from cultured Phr1 KO neurons develop tangles and tortuous tracts during pathfinding, accompanied by major defects in growth cone morphology [61]. It should be noted, however, that no changes in axon length were observed in cultured explants from Phr1 knockout mice [61], suggesting a role for Phr1 in controlling axon guidance rather than outgrowth. Again, DLK has been proposed to be downstream of Phr1 in regulating axon guidance, as loss of Phr1 results in DLK expansion from the growth cone into the dendritic shaft [61]. This would suggest that Phr1 degrades DLK in order to limit its effects during axon outgrowth, possibly through DLK-dependent control of microtubule stability via the downstream MAPK signaling pathway. Other studies, however, saw no change in DLK content in Phr1 KO animals and found that mutation of DLK does not rescue the Phr1 axon outgrowth phenotype, strongly indicating that DLK is not Phr1's primary downstream effector [57]. Furthermore, mutation of Phr1 in zebrafish does not change the activation state of p38, the MAPK downstream of DLK [62]. The same study, however, did find that stabilizing microtubules with nocodazole rescues the tangles and tortuous axon paths in Phr1 mutant cultures, supporting the hypothesis that regulation of microtubule dynamics may lie downstream of Phr1 [62].

Finally, PAM in humans has been shown to associate directly with the atypical F-box protein Fbxo45, a component of the SCF E3 complex [63]. Fbxo45 knockout mouse pups display extensive defects in the morphology of the spinal cord and the thalamocortical, internal capsule, and anterior commissure axon tracts [63]. These diverse studies strongly support a role for PHR E3 ligases in regulating axon guidance, primarily through control of the cytoskeleton.

In addition to the vast literature on the PHR proteins, regulation of axon guidance has been documented for the Rac1 inhibitor, chimaerin, which has recently been shown to be ubiquitinated and degraded in a protein kinase C-dependent manner [64]. Both *α*1- and *α*2-chimaerin splice forms interact with the EphB1 [65] and EphA4 [65–68] receptors, possibly in a phosphorylation-dependent manner [66, 68], although this detail remains controversial [67]. Association with EphA4 stimulates *α*2-chimaerin's GTPase activating (GAP) activity, thereby repressing the activity of Rac1 [66–68], while ephrinA1-dependent growth cone collapse is attenuated by *α*2-chimaerin knockdown [68] or knockout [65, 66]. Given that a wide range of studies of *α*2-chimaerin knockout mice describe aberrant axon guidance and impaired growth cone sensitivity to ephrin signaling, it is likely that the proteasome regulates axon guidance in part through controlling *α*-chimaerin levels at the growth cone, in turn regulating Eph receptor-dependent axon guidance.

## 4. Dendritic Morphogenesis and Arborization by the UPS

Notably, many of the E3 ligases that are important for axon growth and guidance also regulate dendritic outgrowth and arborization, albeit by strikingly different mechanisms. Perhaps the most intriguing example of this is the APC. Whereas nuclear APC-Cdh1 restrains axon outgrowth, cytosolic APC-Cdc20 activity positively regulates dendrite length without changing axon length [69]. Cdc20 knockdown-dependent reductions in dendrite length are rescued through reintroduction of centrosomally targeted PACT-Cdc20, but not with nuclearly targeted NLS-Cdc20. Cdc20 localization at the centrosome is regulated by calcium influx from TRPC5 channels, thereby inducing CamKII*β*-dependent phosphorylation of Cdc20 [70, 71]. Phosphorylation by CaMKII*β* in turn causes the dispersal of Cdc20 from the centrosome, preventing its activation of the APC [70]. Knockdown of either TRPC5 or CaMKII*β* increases dendrite outgrowth in a Cdc20-dependent manner, illustrating another example of calcium-dependent regulation of the proteasome in controlling neurite outgrowth [70, 71].

 Similarly to Cdh1-APC, the effects of Cdc20-APC on dendrite outgrowth depend on the ubiquitination and proteasome-dependent degradation of the transcription factor Id1 [69]. Knockdown of Cdc20 results in an increase in Id1 levels, while knockdown of Id1 is correlated with an increase in dendrite length which is epistatic to Cdc20 knockdown. In yet another layer of regulation, the deubiquitinase activity of USP44 and ubiquitin-binding and stabilization activity of HDAC6 work to balance the ubiquitination state of Cdc20. Rather than causing its degradation, tagging Cdc20 with ubiquitin activates its ability to stimulate the APC at the centrosome. These data support an elegant model whereby the ubiquitination of Cdc20-APC positively regulates its activity, thereby stimulating dendrite outgrowth by ubiquitinating and causing the degradation of Id1. This does, however, leave unanswered precisely how the *cytosolic* ubiquitination of a transcription factor such as Id1 regulates dendrite outgrowth, a process which suggests that Id1 may serve other nontranscriptional functions.

A second E3 ligase that has been shown to regulate both axons and dendrites is Nedd4-1, and, as with Cdc20-APC, the role of Nedd4-1 in dendritic arborization is quite different from its control of axon outgrowth. Knockout of Nedd4-1 in cultured neurons increases dendritic arborization and total dendrite length [72], probably through disabling Nedd4-dependent ubiquitination of the GTPase Rap2A, although ubiquitin chain-specific staining indicates multiple monoubiquitination rather than the polyubiquitination associated with proteasomal degradation [72]. In support of this model, knockdown of one of Rap2A's downstream targets, the actin cytoskeleton regulatory kinase TNIK, phenocopies the reduction in dendritic arborization seen in Nedd4-1 KO cultures [72]. This study suggests that Nedd4-1 is responsible for stimulating dendrite outgrowth by regulating the actin cytoskeleton through targeting the downstream kinases of Rap2A.

While the E3 ligase Ube3A is widely known for its role in the Prader-Willi and Angelman syndromes, rare forms of mental retardation, Ube3A has also recently been implicated in regulation of dendritic arbor growth in fruit flies. Knockdown or mutation of Drosophila Ube3A causes reductions in dendritic arborization [73]. In a rare finding, the authors also identified the E2 which interacts with Ube3A to regulate dendritic arborization as UbcD1. Strikingly, overexpression of Ube3A also reduces dendritic complexity, highlighting the importance of balancing Ube3A abundance in neurons [73].

Another component of the UPS, the COP9 signalosome (CSN), has also been shown to play a role in dendritic morphogenesis in Drosophila. A highly conserved protease complex, the CSN inhibits the assembly of a class of E3 ligases known as Cullin RING ligases through deneddylation of the Cullin scaffold, which causes disassembly of the Cullin RING complex [74]. As the Cullin scaffold promotes the association of an E2 with its target protein, the CSN may regulate specificity of ubiquitination by selectively inhibiting the assembly of specific RING E3 ligases. Loss of CSN function or mutation of associated E3 complex proteins results in an unusually bimodal response in subpopulations of sensory neurons, showing either a significant increase *or* reduction in dendritic arborization [75]. Loss of Cul1 or of the F-box protein Slimb represses arborization, while loss of Cul3 enhances it, suggesting that the Cul1 and Cul3 E3 complexes target proteins with markedly different roles in dendritogenesis. One of the proposed targets for the Cul3 complex is Kelch, an actin cross-linking protein [76]. Mutation of Cul3 increases levels of Kelch protein, while mutation of Kelch reduces dendritic arborization and represses the Cul3 increased arborization phenotype [76], further establishing the complex link between the proteasome and the cytoskeleton.

Another component of an SCF E3 complex, the F-box protein Fbxw8, has been similarly implicated in dendritogenesis. RNAi knockdown of either Fbxw8 or its associated cullin (Cul7) not only reduces dendrite length, but also causes abnormally dispersed Golgi stacks [77]. Intriguingly, RNAi against OBSL1, the cytoskeletal adaptor protein which localizes the Cul7 E3 complex to the Golgi apparatus, causes a similar reduction in dendrite length to direct knockdown of Fbxw8 or Cul7 [77], highlighting the importance of compartment-specific localization to proper UPS function. The same group found that Grasp65, a protein which regulates Golgi morphology and transport, is ubiquitinated and degraded by the Cul7-Fbxw8 complex and that RNAi against Grasp65 blocks the reduction in dendritic branching observed with Cul7 complex knockdown [77].

As with so many other examples, *α*-chimaerin regulates not only axon guidance, but also dendritic arborization. Surprisingly, overexpression of *α*1-chimaerin reduces dendrite length and branching in cerebellar slices, while overexpression of its alternate splice form *α*2-chimaerin increases dendrite length, but not complexity [78]. This differential effect may be due to the SH2 domain present in *α*2-chimaerin, but not its shorter *α*1 form.

An extraordinary example of the variability of UPS function in dendritic morphogenesis is the E3 ligase Mind bomb-1 (Mib1). Mib1 has previously been characterized as a major regulator of Notch signaling through stimulating the ubiquitin-dependent endocytosis of the Notch ligand Delta [79, 80]. Mutations to Mib1 impair Notch signaling, and have been shown to cause major neurodevelopmental defects in a variety of systems, including zebrafish [80], Drosophila [79], *Xenopus* [81], and mouse models [82]. On a smaller scale, however, it has been demonstrated that Mib1 also regulates dendritogenesis in a highly tissue-specific manner. In dissociated mouse cortical neurons, overexpression of Mib1 reduces dendrite length and complexity, while Mib1 activity is in turn negatively regulated by kinase Cdk5 [83]. On the other hand, a later study found that overexpression of Mib1 in dissociated mouse hippocampal cells results in increased dendritic arborization, while knocking down Mib1 and overexpressing its repressor microRNA miR-137 both cause reduced dendritic complexity and spine density [84]. These findings highlight that even within the brain, elements of the UPS are sometimes regulated very differently in different neural tissues, making the determination of the function for each element of the UPS a challenge.

Finally, during metamorphosis, the Drosophila nervous system must undergo extensive rewiring, including the destruction of larval dendritic networks. An example of this process is the C4da neuron, which sheds all of its dendrites before growing a new dendritic arbor. This process is largely dependent on the UPS, as loss of function of the proteasome or mutation of the sole ubiquitin-activating E1 results in a significant reduction in dendrite shedding [85]. This shedding is mediated by the Drosophila caspase Dronc (caspase 9), which is in turn regulated by ubiquitination through the E2/E3 pair UbcD1 and DIAP1 [86]. As with Cdc20, Dronc ubiquitination by DIAP1 does not appear to result in degradation, as local overexpression or loss of DIAP1 does not affect Dronc abundance [87]. Instead, ubiquitination modulates the incomplete cleavage and activation of Dronc [87], demonstrating yet another nondestructive role for the UPS in modulating neural morphogenesis.

## 5. Regulation of Dendritic Spine Morphogenesis by the UPS

In addition to regulating the gross morphology of axons and dendrites, the UPS plays a key role in regulating the morphology of dendritic spines, microscopic protrusions from dendrites that serve as the primary site of excitatory synapses in the mammalian central nervous system. While a number of E3 ligases have been implicated in the regulation of spine morphology and spine density, none have yet been specifically linked with regulating spine outgrowth or retraction.

 The E3 ligase most widely recognized in the regulation of spine density is Ube3A, also known as E6-AP. Loss of maternal Ube3A in transgenic mice causes a reduction in spine density and spine length [88, 89], accompanied by a reduction in spontaneous postsynaptic current frequency [89]. This loss of spines and synapses could be caused by the loss of Ube3A deregulating the abundance of the AMPA receptor trafficking protein Arc, resulting in a severely reduced pool of available AMPA receptors [90]. Another potential cause for Ube3A-related neurodevelopmental dysfunction is that Ube3A also targets Ephexin5, an activator of the small GTPase RhoA, which is associated with the contraction of filamentous actin networks [91]. Overexpression of Ephexin5 reduces spine and synapse density in neuronal cultures, presumably through overactivation of RhoA, while stimulation of EphB2 receptors results in rapid degradation of Ephexin5 [91]. Thus, Ube3A may be able to regulate spine formation through destruction of Ephexin5, thereby removing an inhibitory brake on spinogenesis.

Other elements of the actin cytoskeleton have been shown to be proteasomally regulated, resulting in altered spine density and morphology. Much like Ube3A, the HECT E3 ligase Smurf1 has been shown to regulate RhoA activity through direct ubiquitination and degradation in heterologous cells, resulting in an increase in filopodial protrusions [40], which resemble dendritic filopodia, thought to be precursors of dendritic spines. Similarly, RNAi against the proteasome target *α*1-chimaerin results in reduced spine density and increased filopodial protrusions [78]. This effect is mediated by the interaction between *α*1-chimaerin and the GluN2A subunit of the NMDA receptor, which may direct *α*1-chimaerin to sites of synaptic activity [92].

 While all these data point to a vital role of the UPS in regulating spine density, direct experimental evidence distinguishing between a role for the proteasome in the regulation of spine outgrowth or spine retraction was lacking until very recently, when it was shown that pharmacological inhibition of the proteasome acutely decreases the rate of activity-dependent new spine outgrowth in hippocampal slice cultures [93]. Through sparse transfection of cells in slice culture with Rpt6-S120A, a mutant proteasome subunit which prevents CaMKII-dependent activation of the proteasome [94], the role of the proteasome in spine outgrowth was shown to be postsynaptic and cell autonomous [93]. Finally, mutations in the GluN2B subunit of the NMDA receptor that interrupt interaction between CaMKII and the NMDA receptor [95] were shown to completely block activity-dependent spine outgrowth [93]. Notably, this interaction is also required for the activity-dependent recruitment of CaMKII to synapses [95], which potentially alters proteasome trafficking [16]. Thus, spine outgrowth could serve as an example of a vital neuronal process that is regulated by the UPS in response to rapid, activity-dependent control of both proteasome activity and localization.

## 6. Conclusion and Future Directions

**Figure 2:**
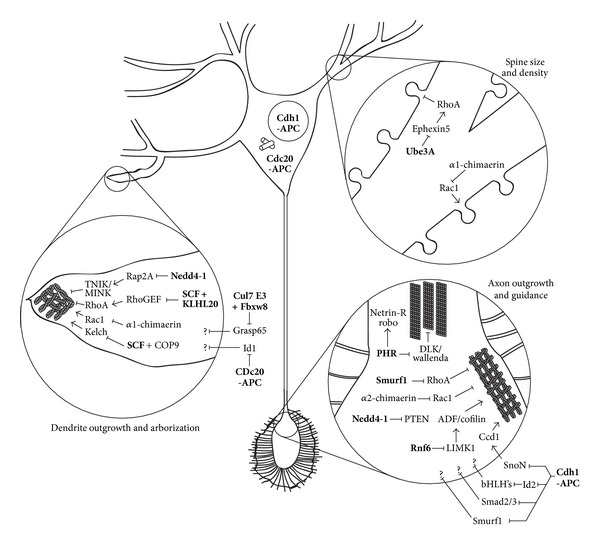
The ubiquitin-proteasome system in regulation of neural morphogenesis. Known signaling cascades for ubiquitin-dependent regulation of the formation of axons (bottom right inset), dendrites (left inset), and dendritic spines (top right inset). Regulatory factors depicted outside of the insets represent signaling from the cell body, rather than local regulation. E3 ligases are depicted in bold; arrows represent positive regulation; bars represent inhibition or degradation.

An ever increasing body of literature has provided tantalizing hints at the intricate control mechanisms by which the UPS regulates the morphogenesis of the neurons most vital processes: axons, dendrites, and dendritic spines (Figure 2). This regulation has spanned multiple modes, including classical proteasome-dependent protein degradation, modification of protein activity and proteasome localization, and the induction of incomplete protein cleavage. Despite remarkable advances over the past decade, several key challenges remain.

 Perhaps the most challenging task is to elucidate the complex mechanisms regulating the ubiquitination state of individual target proteins. The difficulty of this task is highlighted by the complex modes of regulation of Cdc20 [69]. Not only is Cdc20 ubiquitinated in order to activate its effect on the APC, but its ubiquitination state is determined by competing binding partners, seeking to either stabilize or remove its bound ubiquitin moieties. It is not unreasonable to suppose that any of the UPS-targeted proteins discussed could have similar mechanisms at play balancing their ubiquitination, mechanisms that may be just as important for regulating the target protein's stability or activity as the E3 ligases that originally tagged it. Teasing apart these networks may present a veritable cornucopia of options for addressing the disorders associated with aberrant neural morphogenesis.

 Another key challenge is to define how the proteasome is translocated and recruited to specific neuronal compartments. Over the past decade, it has emerged that the proteasome itself is a highly mobile structure; changes in synaptic activity not only alter UPS activity [10, 15, 17], but also cause rapid translocation of the proteasome into the dendrite and dendritic spines [14–16]. Such translocation would provide a means to increase proteasomal degradation in distinct cellular subcompartments and thus to enhance specificity via local regulation of proteasomal activity. In a vast majority of the roles of the proteasome in neural morphogenesis, it acts through the cytoskeleton, and the proteasome itself appears to be bound to the actin cytoskeleton in an activity-dependent manner [15, 94]. Determining the molecular mechanisms that regulate the association of the proteasome with elements of the cytoskeleton may be the key to understanding activity-dependent proteasomal trafficking. In the end, where the garbage disposal is and how it gets there may be just as vital to making a proper neuron as what is going into the garbage.

While the challenges of understanding the role of the UPS in the construction of our brains may be daunting, the potential benefits of finding the answers are considerable. A number of neurological disorders have been associated with reduced proteasome activity and the consequent buildup of ubiquitinated proteins, including Alzheimer's, Huntington's, and Parkinson's [6]. Addressing the role of the proteasome in human neurodegenerative disorders could aid in the development of therapeutics that will help alleviate the suffering associated with these devastating diseases.
